# Improved imputation of low-frequency and rare variants using the UK10K haplotype reference panel

**DOI:** 10.1038/ncomms9111

**Published:** 2015-09-14

**Authors:** Jie Huang, Bryan Howie, Shane McCarthy, Yasin Memari, Klaudia Walter, Josine L. Min, Petr Danecek, Giovanni Malerba, Elisabetta Trabetti, Hou-Feng Zheng, UK10K Consortium, Saeed Al Turki, Antoinette Amuzu, Carl A. Anderson, Richard Anney, Dinu Antony, María Soler Artigas, Muhammad Ayub, Senduran Bala, Jeffrey C. Barrett, Inês Barroso, Phil Beales, Marianne Benn, Jamie Bentham, Shoumo Bhattacharya, Ewan Birney, Douglas Blackwood, Martin Bobrow, Elena Bochukova, Patrick F. Bolton, Rebecca Bounds, Chris Boustred, Gerome Breen, Mattia Calissano, Keren Carss, Juan Pablo Casas, John C. Chambers, Ruth Charlton, Krishna Chatterjee, Lu Chen, Antonio Ciampi, Sebahattin Cirak, Peter Clapham, Gail Clement, Guy Coates, Massimiliano Cocca, David A. Collier, Catherine Cosgrove, Tony Cox, Nick Craddock, Lucy Crooks, Sarah Curran, David Curtis, Allan Daly, Ian N. M. Day, Aaron Day-Williams, George Dedoussis, Thomas Down, Yuanping Du, Cornelia M. van Duijn, Ian Dunham, Sarah Edkins, Rosemary Ekong, Peter Ellis, David M. Evans, I. Sadaf Farooqi, David R. Fitzpatrick, Paul Flicek, James Floyd, A. Reghan Foley, Christopher S. Franklin, Marta Futema, Louise Gallagher, Paolo Gasparini, Tom R. Gaunt, Matthias Geihs, Daniel Geschwind, Celia Greenwood, Heather Griffin, Detelina Grozeva, Xiaosen Guo, Xueqin Guo, Hugh Gurling, Deborah Hart, Audrey E. Hendricks, Peter Holmans, Liren Huang, Tim Hubbard, Steve E. Humphries, Matthew E. Hurles, Pirro Hysi, Valentina Iotchkova, Aaron Isaacs, David K. Jackson, Yalda Jamshidi, Jon Johnson, Chris Joyce, Konrad J. Karczewski, Jane Kaye, Thomas Keane, John P. Kemp, Karen Kennedy, Alastair Kent, Julia Keogh, Farrah Khawaja, Marcus E. Kleber, Margriet van Kogelenberg, Anja Kolb-Kokocinski, Jaspal S. Kooner, Genevieve Lachance, Claudia Langenberg, Cordelia Langford, Daniel Lawson, Irene Lee, Elisabeth M. van Leeuwen, Monkol Lek, Rui Li, Yingrui Li, Jieqin Liang, Hong Lin, Ryan Liu, Jouko Lönnqvist, Luis R. Lopes, Margarida Lopes, Jian'an Luan, Daniel G. MacArthur, Massimo Mangino, Gaëlle Marenne, Winfried März, John Maslen, Angela Matchan, Iain Mathieson, Peter McGuffin, Andrew M. McIntosh, Andrew G. McKechanie, Andrew McQuillin, Sarah Metrustry, Nicola Migone, Hannah M. Mitchison, Alireza Moayyeri, James Morris, Richard Morris, Dawn Muddyman, Francesco Muntoni, Børge G. Nordestgaard, Kate Northstone, Michael C. O'Donovan, Stephen O'Rahilly, Alexandros Onoufriadis, Karim Oualkacha, Michael J. Owen, Aarno Palotie, Kalliope Panoutsopoulou, Victoria Parker, Jeremy R. Parr, Lavinia Paternoster, Tiina Paunio, Felicity Payne, Stewart J. Payne, John R. B. Perry, Olli Pietilainen, Vincent Plagnol, Rebecca C. Pollitt, Sue Povey, Michael A. Quail, Lydia Quaye, Lucy Raymond, Karola Rehnström, Cheryl K. Ridout, Susan Ring, Graham R. S. Ritchie, Nicola Roberts, Rachel L. Robinson, David B. Savage, Peter Scambler, Stephan Schiffels, Miriam Schmidts, Nadia Schoenmakers, Richard H. Scott, Robert A. Scott, Robert K. Semple, Eva Serra, Sally I. Sharp, Adam Shaw, Hashem A. Shihab, So-Youn Shin, David Skuse, Kerrin S. Small, Carol Smee, George Davey Smith, Lorraine Southam, Olivera Spasic-Boskovic, Timothy D. Spector, David St Clair, Beate St Pourcain, Jim Stalker, Elizabeth Stevens, Jianping Sun, Gabriela Surdulescu, Jaana Suvisaari, Petros Syrris, Ioanna Tachmazidou, Rohan Taylor, Jing Tian, Martin D. Tobin, Daniela Toniolo, Michela Traglia, Anne Tybjaerg-Hansen, Ana M. Valdes, Anthony M. Vandersteen, Anette Varbo, Parthiban Vijayarangakannan, Peter M. Visscher, Louise V. Wain, James T. R. Walters, Guangbiao Wang, Jun Wang, Yu Wang, Kirsten Ward, Eleanor Wheeler, Peter Whincup, Tamieka Whyte, Hywel J. Williams, Kathleen A. Williamson, Crispian Wilson, Scott G. Wilson, Kim Wong, ChangJiang Xu, Jian Yang, Gianluigi Zaza, Eleftheria Zeggini, Feng Zhang, Pingbo Zhang, Weihua Zhang, Giovanni Gambaro, J. Brent Richards, Richard Durbin, Nicholas J. Timpson, Jonathan Marchini, Nicole Soranzo

**Affiliations:** 1 The Wellcome Trust Sanger Institute, Wellcome Trust Genome Campus, Hinxton, Cambridge CB10 1HH, UK; 2 Adaptive Biotechnologies Corporation, Seattle Washington 98102, USA; 3 MRC Integrative Epidemiology Unit, School of Social and Community Medicine, University of Bristol, Oakfield House, Oakfield Grove, Clifton, Bristol BS8 2BN, UK; 4 Biology and Genetics, Department of Life and Reproduction Sciences, University of Verona, 37134, Italy; 5 Lady Davis Institute, Jewish General Hospital, Montreal, Quebec, Canada H3T 1E2; 6 Department of Medicine, McGill University, Montreal, Quebec, Canada H3A 1B1; 7 Department of Human Genetics, McGill University, Montreal, Quebec, Canada H3A 1B1; 8 Division of Nephrology and Dialysis, Institute of Internal Medicine, Renal Program, Columbus-Gemelli University Hospital, Catholic University, Rome, Italy; 9 The Department of Twin Research & Genetic Epidemiology, King's College London, St Thomas' Campus, Lambeth Palace Road, London SE1 7EH, UK; 10 Department of Statistics, University of Oxford, 1 South Parks Road, Oxford OX1 3TG, UK; 11 Wellcome Trust Centre for Human Genetics, Roosevelt Drive, Oxford OX3 7BN, UK; 12 Department of Haematology, University of Cambridge, Long Road, Cambridge CB2 0PT, UK; 13 Department of Pathology, King Abdulaziz Medical City, Riyadh, Saudi Arabia; 14 London School of Hygiene and Tropical Medicine, Keppel Street, London WC1E 7HT, UK; 15 Department of Psychiatry, Trinity Centre for Health Sciences, St. James Hospital, James's Street, Dublin 8, Ireland; 16 Genetics and Genomic Medicine and Birth Defects Research Centre, UCL Institute of Child Health, London WC1N 1EH, UK; 17 Departments of Health Sciences and Genetics, University of Leicester, Leicester LE1 7RH, UK; 18 Division of Developmental Disabilities, Department of Psychiatry, Queen's University, Kingston, Canada N6C 0A7; 19 University of Cambridge Metabolic Research Laboratories, and NIHR Cambridge Biomedical Research Centre, Wellcome Trust-MRC Institute of Metabolic Science, Addenbrooke's Hospital, Cambridge CB2 0QQ, UK; 20 Department of Clinical Biochemistry and The Copenhagen General Population Study, Herlev and Gentofte Hospital, Copenhagen University Hospital, Herlev, 2730, Denmark; 21 The Faculty of Health and Medical Sciences, University of Copenhagen, Copenhagen 2200, Denmark; 22 Department of Cardiovascular Medicine and Wellcome Trust Centre for Human Genetics, Roosevelt Drive, Oxford OX3 7BN, UK; 23 European Molecular Biology Laboratory, European Bioinformatics Institute, Wellcome Trust Genome Campus, Hinxton, Cambridge CB10 1SD, UK; 24 Division of Psychiatry, The University of Edinburgh, Royal Edinburgh Hospital, Edinburgh EH10 5HF, UK; 25 Department of Medical Genetics, Cambridge Institute for Medical Research, University of Cambridge CB2 0XY, UK; 26 Department of Child Psychiatry, Institute of Psychiatry, Psychology and Neuroscience, King's College London, 16 De Crespigny Park, London SE5 8AF, UK; 27 NIHR BRC for Mental Health, Institute of Psychiatry, Psychology and Neuroscience and SLaM NHS Trust, King's College London, 16 De Crespigny Park, London SE5 8AF, UK; 28 MRC Social, Genetic and Developmental Psychiatry Centre, Institute of Psychiatry, Psychology and Neuroscience, King's College London, Denmark Hill, London SE5 8AF, UK; 29 North East Thames Regional Genetics Service, Great Ormond Street Hospital NHS Foundation Trust, London WC1N 3JH, UK; 30 Dubowitz Neuromuscular Centre, UCL Institute of Child Health & Great Ormond Street Hospital, London WC1N 1EH, UK; 31 Institute of Cardiovascular Science, University College London, Gower Street, London WC1E 6BT, UK; 32 The Department of Epidemiology and Biostatistics, Imperial College London, St. Mary's campus, Norfolk Place, Paddington, London W2 1PG, UK; 33 Leeds Genetics Laboratory, St James University Hospital, Beckett Street, Leeds, West Yorkshire, LS9 7TF, UK; 34 Department of Epidemiology, Biostatistics and Occupational Health, McGill University, Montreal, Quebec, Canada H3A 1A2; 35 Institut für Humangenetik, Uniklinik Köln, Kerpener Str34, 50931 Köln, Germany; 36 Institute for Maternal and Child Health–IRCCS Burlo Garofolo–Trieste, University of Trieste, 34137 Trieste, Italy; 37 Department of Medical, Surgical and Health Sciences, University of Trieste, 34100 Trieste, Italy; 38 Lilly Research Laboratories, Eli Lilly & Co. Ltd., Erl Wood Manor, Sunninghill Road, Windlesham, Surrey, GU20 6PH, UK; 39 MRC Centre for Neuropsychiatric Genetics & Genomics, Institute of Psychological Medicine & Clinical Neurosciences, School of Medicine, Cardiff University, Cardiff CF14 4XN, UK; 40 Sheffield Diagnostic Genetics Service, Sheffield Childrens' NHS Foundation Trust, Western Bank, Sheffield S10 2TH, UK; 41 University of Sussex, Brighton BN1 9RH, UK; 42 Sussex Partnership NHS Foundation Trust, Swandean, Arundel Road, Worthing, West Sussex, BN13 3EP, UK; 43 University College London (UCL), UCL Genetics Institute, Darwin Building, Gower Street, London WC1E 6BT, UK; 44 Bristol Genetic Epidemiology Laboratories, School of Social and Community Medicine, University of Bristol, Oakfield House, Oakfield Grove, Clifton, Bristol BS8 2BN, UK; 45 Computational Biology & Genomics, Biogen Idec, 14 Cambridge Center, Cambridge, Massachusetts 02142, USA; 46 Department of Nutrition and Dietetics, School of Health Science and Education, Harokopio University, Athens 17671, Greece; 47 Department of Medical and Molecular Genetics, Division of Genetics and Molecular Medicine, King's College London School of Medicine, Guy's Hospital, London SE1 9RT, UK; 48 BGI-Shenzhen, Shenzhen 518083, China; 49 Genetic Epidemiology Unit, Department of Epidemiology, Erasmus MC, Rotterdam 3000 CA, Netherlands; 50 University College London (UCL) Department of Genetics, Evolution & Environment (GEE), Gower Street, London WC1E 6BT, UK; 51 University of Queensland Diamantina Institute, Translational Research Institute, Brisbane, Queensland, 4102, Australia; 52 MRC Human Genetics Unit, MRC Institute of Genetics and Molecular Medicine, at the University of Edinburgh, Western General Hospital, Edinburgh EH4 2XU, UK; 53 The Genome Centre, John Vane Science Centre, Queen Mary, University of London, Charterhouse Square, London EC1M 6BQ, UK; 54 Cardiovascular Genetics, BHF Laboratories, Rayne Building, Institute of Cardiovascular Sciences, University College London, London WC1E 6JJ, UK; 55 Experimental Genetics Division, Sidra, P.O. Box 26999 Doha, Qatar; 56 UCLA David Geffen School of Medicine, Los Angeles, California 90095, USA; 57 Department of Oncology, McGill University, Montreal, Quebec, Canada H2W 1S6; 58 HeLEX—Centre for Health, Law and Emerging Technologies, Nuffield Department of Population Health, University of Oxford, Old Road Campus, Oxford OX3 7LF, UK; 59 Department of Biology, University of Copenhagen, Ole Maaløes Vej 5, DK-2200 Copenhagen, Denmark; 60 University College London (UCL), Molecular Psychiatry Laboratory, Division of Psychiatry, Gower Street, London WC1E 6BT, UK; 61 Department of Mathematical and Statistical Sciences, University of Colorado, Denver, Colorado 80202, USA; 62 Human Genetics Research Centre, St George's University of London SW17 0RE, UK; 63 Department of Quantitative Social Science, UCL Institute of Education, University College London, 20 Bedford Way, London WC1H 0AL; 64 Analytic and Translational Genetics Unit, Massachusetts General Hospital, Boston Massachusetts 02114, USA; 65 Program in Medical and Population Genetics, Broad Institute of Harvard and MIT, Cambridge Massachusetts 02142, USA; 66 National Cancer Research Institute, Angel Building, 407 St John Street, London EC1V 4AD, UK; 67 Genetic Alliance UK, 4D Leroy House, 436 Essex Road, London N1 3QP, UK; 68 SW Thames Regional Genetics Lab, St George's University, Cranmer Terrace, London SW17 0RE, UK; 69 Vth Department of Medicine, Medical Faculty Mannheim 68167, Germany; 70 National Heart and Lung Institute, Imperial College London, London W12 0NN, UK; 71 MRC Epidemiology Unit, University of Cambridge School of Clinical Medicine, Box 285, Institute of Metabolic Science, Cambridge Biomedical Campus, Cambridge CB2 0QQ, UK; 72 Schools of Mathematics and Social and Community Medicine, University of Bristol, Oakfield House, Oakfield Grove, Clifton, Bristol BS8 2BN, UK; 73 Behavioural and Brain Sciences Unit, UCL Institute of Child Health, London WC1N 1EH, UK; 74 BGI-Europe, London EC2M 4YE, UK; 75 National Institute for Health and Welfare (THL), Helsinki FI-00271, Finland; 76 Cardiovascular Centre, University of Lisbon, Portugal; 77 Illumina Cambridge Ltd, Chesterford Research Park, CB10 1XL, UK; 78 National Institute for Health Research (NIHR) Biomedical Research Centre at Guy's and StThomas' Foundation Trust, London SE1 9RT, UK; 79 Clinical Institute of Medical and Chemical Laboratory Diagnostics, Medical University of Graz, Graz 8036, Austria; 80 Synlab Academy, Synlab Services GmbH, Mannheim, Germany; 81 Medical Clinic V (Nephrology, Hypertensiology, Rheumatology, Endocrinolgy, Diabetology), Mannheim Medical Faculty, Heidelberg University, Mannheim, 68167, Germany; 82 Department of Genetics, Harvard Medical School, Boston, Massachusetts 02115, USA; 83 The Patrick Wild Centre, The University of Edinburgh, Edinburgh EH10 5HF, UK; 84 Department of Medical Sciences, University of Torino, 10124, Italy; 85 Institute of Health Informatics, Farr Institute of Health Informatics Research, University College London (UCL), 222 Euston Road, London NW1 2DA, UK; 86 School of Social and Community Medicine, Canynge Hall, 39 Whatley Road, Bristol BS8 2PS, UK; 87 Department of Mathematics, Université de Québec À Montréal, Montréal, Québec, Canada H3C 3P8; 88 Institute for Molecular Medicine Finland (FIMM), University of Helsinki, Helsinki FI-00014, Finland; 89 Institute of Neuroscience, Henry Wellcome Building for Neuroecology, Newcastle University, Framlington Place, Newcastle upon Tyne NE2 4HH, UK; 90 University of Helsinki, Department of Psychiatry, Helsinki FI-00014, Finland; 91 North West Thames Regional Genetics Service, Kennedy-Galton Centre, Northwick Park Hospital, Watford Road, Harrow HA1 3UJ, UK; 92 Connective Tissue Disorders Service, Sheffield Diagnostic Genetics Service, Sheffield Children's NHS Foundation Trust, Western Bank, Sheffield S10 2TH, UK; 93 Molecular Genetics, Viapath at Guy's Hospital, London SE1 9RT, UK; 94 ALSPAC & School of Social and Community Medicine, University of Bristol, Oakfield House, Oakfield Grove, Clifton, Bristol BS8 2BN, UK; 95 Human Genetics Department, Radboudumc and Radboud Institute for Molecular Life Sciences (RIMLS), Geert Grooteplein 25, Nijmegen, 6525 HP, The Netherlands; 96 Department of Clinical Genetics, Great Ormond Street Hospital, London, WC1N 3JH, UK; 97 Clinical Genetics, Guy's & St Thomas' NHS Foundation Trust, London SE1 9RT, UK; 98 Institute of Medical Sciences, University of Aberdeen, AB25 2ZD, UK; 99 School of Oral and Dental Sciences, University of Bristol, Lower Maudlin Street, Bristol BS1 2LY, UK; 100 School of Experimental Psychology, University of Bristol, 12a Priory Road, Bristol BS8 1TU, UK; 101 National Institute for Health Research (NIHR) Leicester Respiratory Biomedical Research Unit, Glenfield Hospital, Leicester LE3 9QP, UK; 102 Division of Genetics and Cell Biology, San Raffaele Scientific Institute, Milan 20132, Italy; 103 Department of Clinical Biochemistry KB3011, Rigshospitalet, Copenhagen University Hospital, Blegdamsvej 9, DK-2100 Copenhagen, Denmark; 104 Maritime Medical Genetics Service, 5850/5980 University Avenue, PO Box 9700, Halifax, Nova Scotia, Canada B3K 6R8; 105 Queensland Brain Institute, University of Queensland, Brisbane, Queensland 4072, Australia; 106 Princess Al Jawhara Albrahim Center of Excellence in the Research of Hereditary Disorders, King Abdulaziz University, Jeddah, Saudi Arabia; 107 Macau University of Science and Technology, Avenida Wai long, Taipa, Macau 999078, China; 108 Department of Medicine and State Key Laboratory of Pharmaceutical Biotechnology, University of Hong Kong, 21 Sassoon Road, Hong Kong; 109 Population Health Research Institute, St George's University of London, London SW17 0RE, UK; 110 The Centre for Translational Omics – GOSgene, UCL Institute of Child Health, London WC1N 1EH, UK; 111 School of Medicine and Pharmacology, University of Western Australia, Perth, WA 6009, Australia; 112 Department of Endocrinology and Diabetes, Sir Charles Gairdner Hospital, Nedlands, WA 6009, Australia; 113 Renal Unit, Department of Medicine, University of Verona, 37126, Verona, Italy

## Abstract

Imputing genotypes from reference panels created by whole-genome sequencing (WGS) provides a cost-effective strategy for augmenting the single-nucleotide polymorphism (SNP) content of genome-wide arrays. The UK10K Cohorts project has generated a data set of 3,781 whole genomes sequenced at low depth (average 7x), aiming to exhaustively characterize genetic variation down to 0.1% minor allele frequency in the British population. Here we demonstrate the value of this resource for improving imputation accuracy at rare and low-frequency variants in both a UK and an Italian population. We show that large increases in imputation accuracy can be achieved by re-phasing WGS reference panels after initial genotype calling. We also present a method for combining WGS panels to improve variant coverage and downstream imputation accuracy, which we illustrate by integrating 7,562 WGS haplotypes from the UK10K project with 2,184 haplotypes from the 1000 Genomes Project. Finally, we introduce a novel approximation that maintains speed without sacrificing imputation accuracy for rare variants.

## Abstract (web summary)

Imputation uses genotype information from SNP arrays to infer the genotypes of missing markers. Here, the authors show that an imputation reference panel derived from whole-genome sequencing of 3,781 samples from the UK10K project improves the imputation accuracy and coverage of low frequency variants compared to existing methods.

## Graphical abstract

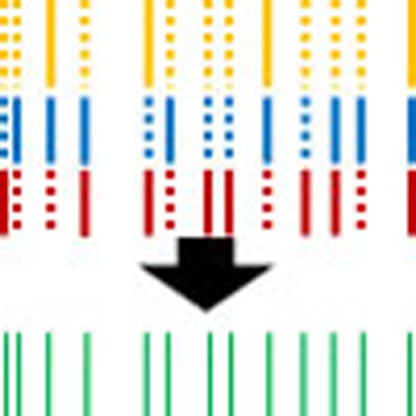

Statistical inference of missing genotypes (imputation), where genotyped markers from SNP arrays are used to impute unobserved genotypes from haplotype panels such as the HapMap data, has been instrumental to the discovery of thousands of complex trait loci in meta-analyses of genome-wide association studies (GWAS)^1^,^2^. Whole-genome sequencing (WGS) provides near-complete characterization of genetic variation, but it is still prohibitive for researchers to conduct WGS on the large number of samples that are needed to study phenotypic associations of low-frequency and rare genetic variants (minor allele frequency (MAF) <1–5% and <1% respectively). Recently, the 1000 Genomes Project (1000GP) has provided phased haplotypes for more than a thousand samples from diverse worldwide populations, thereby boosting variant coverage and imputation quality, particularly for variants with MAFs of 1–5% (ref. 3). Imputation using this large reference panel has been made computationally efficient by pre-phasing of GWAS samples^4^ and approximations that select a subset of reference haplotypes^5^.

Here we describe a novel WGS imputation panel comprising 3,781 samples from the UK10K Cohorts project^6^. We show that this reference panel greatly increases accuracy and coverage of low-frequency variants relative to a panel of 1,092 individuals from the 1000GP. In addition, we show that imputation accuracy can improve substantially when reference haplotypes are re-phased after initial WGS genotype calling. We present a practical solution for combining imputation reference panels to increase variant coverage, and we introduce a new approximation that maintains the speed of existing approximations while achieving higher accuracy.

## Results

### The UK10K imputation panel

The UK10K Cohorts Project^6^ includes two population samples from the UK (http://www.uk10k.org/studies/cohorts.html). The TwinsUK registry comprises unselected, mostly female volunteers ascertained from the general population through national media campaigns in the UK^7^. The Avon Longitudinal Study of Parents and Children (ALSPAC) is a population-based birth cohort study that recruited >13,000 pregnant women resident in Bristol (formerly Avon), UK^8^. A total of 1,990 individuals from TwinsUK and 2,040 individuals from ALSPAC were consented for sequencing. Variant sites and genotype likelihoods were called using SAMtools^9^, and genotypes were refined and phased using Beagle^10^, following similar procedures to the 1000GP (Methods)^3^. After QC, 45,492,035 variant sites (42,001,210 single-nucleotide variants and 3,490,825 insertion/deletions (INDELs)) were retained (Table 1) in 1,854 and 1,927 individuals in the TwinsUK and ALSPAC panels, respectively. We downloaded phased haplotypes from 1000GP (Phase 1 integrated v3), which include a total of 39,527,072 sites. We developed new software functionality for merging haplotype reference panels (Supplementary Note 1 and Supplementary Fig. 1). For imputation using the merged panel, here we removed multi-allelic sites and further excluded variants seen only in 1000GP or seen only once in the combined 1000GP+UK10K data set (singletons, see footnote of Table 1 for details). The choice of removing 1000GP-only and singleton sites was designed to specifically evaluate the impact of the increased European-ancestry panel in UK10K vis-à-vis the smaller 1000GP EUR panel. A total of 26,032,603 sites were retained for the imputation reference panel of UK10K panel, and 32,449,428 sites for the imputation reference panel of 1000GP. Given that 16,122,337 sites exist in both panels, combining the two reference panels results in a total of 42,359,694 sites. Overall, 5,775,752 (35.8%) of the overlapping sites had frequencies >5% and another 2,451,738 (15.2%) had frequencies between 1 and 5% in the UK10K sample.

### Imputation evaluation of UK10K versus 1000GP

As a first assessment of the UK10K reference panel, we performed a leave-one-out cross-validation on a pseudo-GWAS of UK ancestry, corresponding to a sub-sample of 1,000 individuals from the UK10K WGS data set (500 from TwinsUK and 500 from ALSPAC). For this experiment, we removed each sample from the reference panel in turn, selected 13,413 sites on chromosome 20 from the Illumina 610 k bead chip (pseudo-GWAS panel), and imputed all other sites on this chromosome from a given reference panel. We conducted the imputation with three haplotype reference panels: the 1000GP Phase 1 panel, the ‘original' UK10K panel produced by initial genotype refinement and haplotyping with BEAGLE, and a ‘re-phased' UK10K panel that was generated by using SHAPEIT v2 (ref. 11) to estimate haplotypes from the BEAGLE genotypes (Supplementary Fig. 2). The accuracy of imputed variants was calculated as the squared Pearson correlation coefficient (*r*^2^) between imputed genotype dosages in (0–2) and masked sequence genotypes in (0,1,2). The results were stratified into non-overlapping MAF bins for plotting.

**Figure 1:**
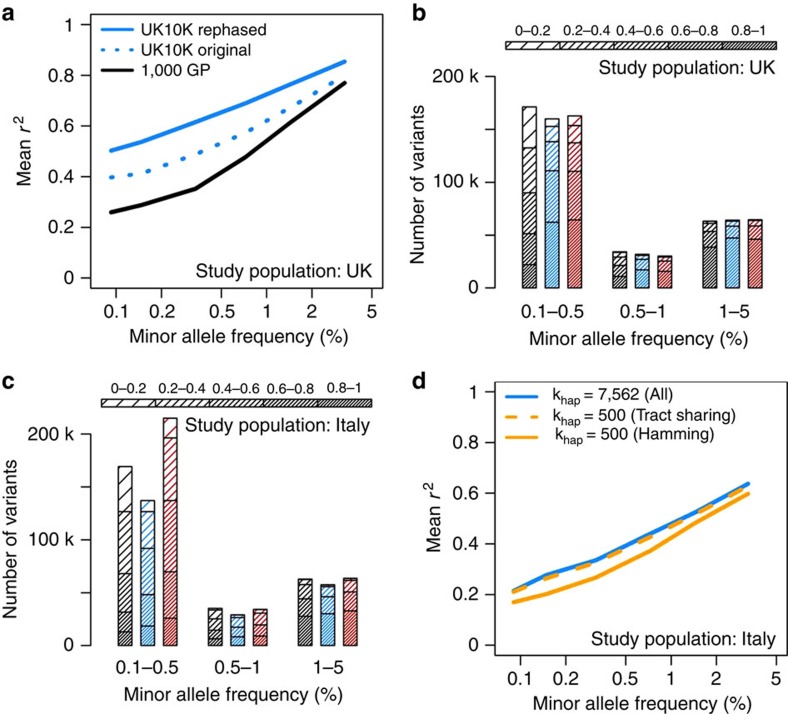
Imputation performance for different imputation strategies and reference panels. (**a**) Imputation accuracy in the UK10K pseudo-GWAS test panel using reference panels from 1000GP (black) and UK10K (blue). The ‘original' UK10K reference panel (dotted blue line) was produced by standard genotype refinement of low-coverage sequencing data, whereas the ‘rephased' reference panel (solid blue line) was produced by running SHAPEIT v2 on the genotypes called by BEAGLE to improve haplotype accuracy. (**b**) Number of imputed variants in UK10K pseudo-GWAS panel as a function of predicted minor allele frequency in the study cohort (*x*-axis), expected imputation *r*^2^ (density of shading), and reference panel: 1000GP (black), UK10K (blue), or combined UK10K and 1000GP (red). Confidently imputed variants are shown in the bottom segment of each bar for easy comparison. Note that expected *r*^2^ tends to be larger than true *r*^2^. (**c**) As in **b**, but using the INCIPE cohort (representative of the general Italian population) as a pseudo-GWAS panel. (**d**) Imputation accuracy in the INCIPE pseudo-GWAS panel using the UK10K reference panel and different imputation approximations. Results are provided for a run that used all reference haplotypes with no approximation (blue solid line), a run that used an established Hamming distance approximation (orange solid line), and a run that used a new tract sharing approximation (orange dashed line).

The results of this experiment are shown in Fig. 1a, which focuses on variants with MAF<5%. The corresponding plot for all MAF is shown in Supplementary Fig. 3. Both UK10K reference panels (blue dotted and solid lines) produced higher accuracy than the 1000GP panel (black line), with greater gains at lower frequencies. These trends were expected due to the larger sample size and better ancestry matching of the UK10K reference panel to the pseudo-GWAS data. Notably, the UK10K reference panel yielded much higher imputation accuracy after re-phasing with SHAPEIT v2 (solid versus dotted blue lines): the mean *r*^2^ at low frequencies increased by >0.1 (20%) after re-phasing, which implies a substantial boost in the power to detect associations. A large imputation panel is a resource that can inform a variety of association studies, so these results suggest that taking the time to improve a WGS panel's haplotype quality could have substantial downstream benefits.

### Evaluation of combining two reference panels

It is becoming increasingly common for investigators to conduct their own WGS of particular study populations, and a natural goal is to combine these data sets with publicly available reference panels (such as 1000GP) to increase sample size and variant coverage for imputation of GWAS cohorts. This is already a ubiquitous problem, and there are multiple ways to integrate WGS data sets that require different levels of data sharing and computing power. In this work, we suggest a simple approach that should be feasible for most groups that have sufficient computational resources for GWAS imputation. Our approach is to take two-phased reference panels and reciprocally impute them up to the union set of variants, then use this combined panel for GWAS imputation; we have implemented this functionality in IMPUTE2 (ref. 1) (details are shown in Supplementary Table 1 and Supplementary Note 1).

To evaluate this new functionality, we used a combined 1000GP+UK10K panel to perform imputation with pseudo-GWAS data sets drawn from the UK and Italy (details below). In each of these comparisons, we imputed all available reference variants and stratified them by expected *r*^2^, which is a confidence metric produced by IMPUTE2 (also known as ‘info' in the software output). Unlike the true *r*^2^ metric, which is usually calculated by masking and imputing ‘truth' genotypes, the expected *r*^2^ metric allows direct comparisons of reference panel performance across study populations that have substantially different sets of genotyped truth variants. We have found that predicted *r*^2^ values tend to be larger than true *r*^2^ values for low-frequency variants (for example, only ∼2/3 of variants with expected *r*^2^≥0.4 and MAF<5% have true *r*^2^≥0.4), so the absolute numbers of high-confidence imputed variants reported in this section should be treated as upper bounds; the emphasis is on qualitative patterns between reference panels and between study populations.

Figure 1b shows how a combined 1000GP+UK10K panel (red) produced by this method performed against each panel separately (1000GP, black; UK10K, blue) when imputing a pseudo-GWAS of UK ancestry. For these evaluations, we used UK10K and 1000GP haplotype panels rephased using SHAPEIT v2, which were previously shown to yield more accurate imputation compared with the corresponding ‘original' haplotypes. The combined and UK10K panels produced very similar numbers of high-confidence (expected *r*^2^>0.8) variants at MAFs of 0.5% and higher, implying that the combined panel is neither helpful nor harmful for imputing common and low-frequency variants when a large, population-specific panel is available. On chromosome 20, the combined panel added 2,356 high-confidence rare variants that were not captured by the UK10K panel (MAF<0.5%; 4% increase), which could reflect mutations that have drifted to very low frequencies in the UK but persist on the same haplotype background elsewhere in Europe^5^,^12^.

Figure 1c provides the results of a similar evaluation carried out in a population in northern Italy (INCIPE cohort), also based on chromosome 20. The INCIPE cohort was newly genotyped in this study, using Illumina HumanCoreExome-12v1-1 arrays. After stringent QC (Online Supplementary Methods), chromosome 20 genotypes from 6,300 SNPs in 2,145 participants were used to drive imputation with each reference panel. In this data set the UK10K reference panel outperformed the 1000GP panel in all frequency bins, despite the fact that the 1000GP includes a panel (TSI, or ‘Toscani in Italia') that is genetically more similar to the study population. This confirms previous findings^13^ that reference sample size is often more important than population matching. As before, the combined 1000GP+UK10K panel yielded a larger number of high-confidence imputed variants than the UK10K panel alone—here, the combined panel added 7,466 well-imputed variants with MAF<0.5%, for a 40% increase in rare variants over the UK10K panel (Fig. 1b). These results suggest that it can be especially useful to combine the strengths of multiple panels when a large, population-specific reference set is not available for a particular GWAS population.

### Imputation metrics for choosing reference haplotypes

In the course of our analyses, we noticed that some rare variants were imputed well when using the entire UK10K reference panel to drive imputation, yet poorly when using IMPUTE2's *k*_*hap*_ approximation (all of the results described above are based on using the full reference panel). This approximation reduces the computational cost of imputation by using a region-wide (for example, across a 3MB imputation chunk) Hamming distance metric to reduce the number of reference haplotypes used by a given GWAS haplotype (see also Supplementary Fig. 4). Our investigation of these variants led us to develop a new approximation that uses local (rather than region-wide) haplotype sharing to choose a subset of reference haplotypes (see Supplementary Note 2 for details). This approximation delivers a speed boost similar to that of the existing *k*_*hap*_ approximation, but it does not sacrifice imputation accuracy at rare and low-frequency variants. For example, Fig. 1d shows the results of imputing the INCIPE pseudo-GWAS data with the UK10K reference panel (see also Supplementary Fig. 5). The full UK10K panel produced the highest accuracy (solid blue line), whereas the *k*_*hap*_ approximation based on Hamming distance (solid orange line) was less accurate for SNPs with MAF<5%. By contrast, our new approximation based on haplotype tract sharing (dashed orange line) was nearly as accurate as the full reference panel, at ∼10% of the computing time (see also Supplementary Fig. 6). All of these strategies for choosing reference haplotypes improved slightly (1–5% increase in mean *r*^2^) when the 1000GP haplotypes were added to the UK10K panel, but their relative accuracies remained similar to those shown in Fig. 1d. Further speed improvements are possible for a modest price in accuracy (see Supplementary Note 2).

## Discussion

As WGS becomes a standard tool for population and disease genetics, there will be many questions about how to design sequencing studies, how to process the data, how to combine data across studies, and how to limit the computational costs of downstream analysis. With data from one of the most ambitious population sequencing studies to date, we have demonstrated the value of a large, UK-specific reference panel for imputation in British cohorts and in other European populations. Our results show that state-of-the-art phasing methods like SHAPEIT v2 are essential for creating high-quality haplotype panels. Combining WGS data across studies is a desirable goal, and we have implemented an approach in IMPUTE2 that can integrate sets of phased haplotypes to produce a unified reference panel; other strategies for combining WGS data may improve haplotype quality, but our approach has the advantage of being relatively simple and fast. Finally, we have proposed a new approximation that will help reduce the trade-off between imputation speed and accuracy as reference panels continue to grow. The novel strategies we have presented will inform other investigators who wish to use WGS reference panels for imputation, and they will spur additional methods development as population sequencing resources proliferate.

Future efforts to combine multiple large low-coverage sequencing datasets into a substantially larger haplotype resource will likely increase imputation performance, especially at variants with frequencies below 0.1%. We generated a combined reference panel with 42.4 million imputable sites, which is much larger than the 26.6 million imputable sites in the UK10K panel or 32.5 million imputable sites in the 1000GP panel. The UK10K WGS haplotypes for 3,781 samples are available for download from the European Genome-phenome Archive (https://www.ebi.ac.uk/ega/) under managed access conditions (http://www.uk10k.org/data_access). The functionality described in this work is available from the IMPUTE2 website (http://mathgen.stats.ox.ac.uk/impute/impute_v2.html) and the SHAPEIT v2 website (https://mathgen.stats.ox.ac.uk/genetics_software/shapeit/shapeit.html).

## Methods

### Sample collections

The ALSPAC is a long-term health research project. More than 14,000 mothers enrolled during pregnancy in 1991 and 1992, and the health and development of their children has been followed in great detail ever since^8^. A random sample of 2,040 study participants was selected for WGS. The ALSPAC Genetics Advisory Committee approved the study and all participants gave signed consent to the study.

The Department of Twin Research and Genetic Epidemiology, is the UK's only twin registry of 11,000 identical and non-identical twins between the ages of 16 and 85 years (ref. 14). The database used to study the genetic and environmental aetiology of age-related complex traits and diseases. The St Thomas's Hospital Ethics Committee approved the study and all participants gave signed consent to the study.

### Sequence data production

Low-read depth WGS was performed in the TwinsUK and ALSPAC as part of the UK10K project. Methods for the generation of these data are described in detail as follows^6^:

Low coverage WGS was performed at both the Wellcome Trust Sanger Institute and the Beijing Genomics Institute (BGI). DNA (1–3 μg) was sheared to 100–1,000 bp using a Covaris E210 or LE220 (Covaris, Woburn, MA, USA). Sheared DNA was size subjected to Illumina paired-end DNA library preparation. Following size selection (300–500 bp insert size), DNA libraries were sequenced using the Illumina HiSeq platform as paired-end 100 base reads according to manufacturer's protocol.

Data generated at the Sanger Institute and BGI were aligned to the human reference separately by the respective centres. The BAM files^3^ produced from these alignments were submitted to the European Genome-phenome Archive. The Vertebrate Resequencing Group at the Sanger Institute then performed further processing.

Sequencing reads that failed QC were removed using the Illumina GA Pipeline, and the rest were aligned to the GRCh37 human reference, specifically the reference used in Phase 1 of the 1000GP (ftp://ftp.1000genomes.ebi.ac.uk/vol1/ftp/technical/reference/human_g1k_v37.fasta.gz). Reads were aligned using BWA (v0.5.9-r16) (ref. 4). This involved the following steps:

1. Index the reference fasta file:

bwa index -a bwtsw <reference_fasta>

2. For each fastq file:

bwa aln -q 15 -f <sai_file> <reference_fasta> <fastq_file>

3. Create SAM files [sam] using bwa sampe for paired-end reads:

bwa sampe -f <sam_file> <reference_fasta> <sai_files> <fastq_files>

4. Create sorted BAM from SAM. For alignments created at the Sanger this was done using Picard (v1.36; http://picard.sourceforge.net/) SamFormatConverter and samtools (v0.1.11) sort. For alignments created at the BGI, this was done using samtools (v0.1.8) view and samtools sort.

5. PCR duplicates reads in the Sanger alignments were marked as duplicate using the Picard MarkDuplicates, whereas in the BGI alignments they were removed using samtools rmdup.

Further processing to improve SNP and INDEL calling, including realignment around known INDELs, base quality score recalibration, addition of BAQ tags, merging and duplicate marking follows that used for Illumina low coverage data in Phase 1 of the 1000GP^5^. Software versions used for UK10K for the steps described in that section were GATK version 1.1-5-g6f43284, Picard version 1.64 and samtools version 0.1.16.

SNP and INDEL calls were made using samtools/bcftools (version 0.1.18-r579: https://github.com/samtools/samtools/commit/70c740facc966321754c6bfcc6d61ea056480638)^6^ by pooling the alignments from 3,910 individual low coverage BAM files. All-samples and all-sites genotype likelihood files (bcf) were created with the samtools mpileup command

samtools mpileup -EDVSp -C50 -m3 -F0.2 -d 8000 -P ILLUMINA -g

with the flags:

*C*=Coefficient for downgrading mapping quality for reads containing excessive mismatches.

*d*=At a position, read maximally *d* reads per input BAM

Variants were then called using the following bcftools command to produce a VCF file^7^

bcftools view -m 0.9 -vcgN.

For calling on chromosome X and Y, the following settings were applied. The pseudo-autosomal region (PAR) was masked on chromosome Y in the reference fasta file. Male samples were called as diploid in the PAR on chromosome X, and haploid otherwise. No calls were made on chromosome Y for female samples. Diploid/haploid calls were made using the -s option in bcftools view. The PAR regions were: X-PAR1 (60,001-2,699,520); X-PAR2 (154,931,044-155,260,560); Y-PAR1 (10,001-2,649,520); Y-PAR2 (59,034,050-59,363,566). The pipeline (run-mpileup) used to create the calls is available from https://github.com/VertebrateResequencing/vr-codebase/tree/develop.

The observation of spikes in the insertion/deletion ratio in sequencing cycles of a subset of the sequencing runs were linked to the appearance of bubbles in the flow cell during sequencing. To counteract this, the following post-calling filtering was applied. The bamcheck utility from the samtools package was used to create a distribution of INDELs per sequencing cycle. Lanes with INDELs predominantly clustered at certain read cycles were marked as problematic, specifically where the highest peak was 5x bigger than the median of the distribution. The list of problematic lanes included 159 samples. In the next step we checked mapped positions of the affected reads to see if they overlapped with called INDELs, which they did for 1,694,630 called sites. The genotypes and genotype likelihoods of affected samples were then set to the reference genotype unless there was a support for the indel also in a different, unaffected lane from the same sample. In total, 140,163 genotypes were set back to reference and 135,647 sites were excluded by this procedure. Note that this step was carried out on raw, unfiltered calls prior to Variant Quality Score Recalibration (VQSR) filtering.

VQSR^8^ was used to filter sites. For SNPs, the GATK (version 1.3-21) UnifiedGenotyper was used to recall the sites/alleles discovered by samtools in order to generate annotations to be used for recalibration. Recalibration for the INDELs used annotations derived from the built-in samtools annotations. The GATK VariantRecalibrator was then used to model the variants, followed by GATK ApplyRecalibration, which assigns VQSLOD (variant quality score log odds ratio) values to the variants. For more detailed information on VQSR, see http://www.broadinstitute.org/gsa/wiki/index.php/Variant_quality_score_recalibration. SNPs and INDELs were modeled separately, with parameters given below:

1. Annotations
  - SNPs: QD, DP, FS, MQ, HaplotypeScore, MQRankSum, ReadPosRankSum, InbreedingCoeff
  - INDELSs: MSD, MDV, MSQ, ICF, DP, SB, VDB
2. Training set
  - SNPs: HapMap 3.3: hapmap_3.3.b37.sites.vcf, Omni 2.5M chip: 1000G_omni2.5.b37.sites.vcf
  - INDELs: Mills-Devine, 1000 Genomes Phase I
3. Truth Set
  - SNPs: HapMap 3.3: hapmap_3.3.b37.sites.vcf
  - INDELS: Mills-Devine
4. Known Set
  - SNPs: dbSNP build 132: dbsnp_132.b37.vcf
  - INDELs: Mills-Devine

The truth-set sites are defined as truly showing variation from the reference. VQSLOD scores are calibrated by how many of the truth sites are retained when sites with a VQSLOD score below a given threshold are filtered out. For single-nucleotide variants sites a truth sensitivity of 99.5%, which corresponded to a minimum VQSLOD score of −0.6804 was selected, that is, for this threshold 99.5% of truth sites were retained. For INDEL sites a truth sensitivity of 97%, which corresponded to a minimum VQSLOD score of 0.5939 was chosen. Finally, we also introduced the filter *P*<10^−6^ to remove sites that failed the Hardy–Weinberg equilibrium.

The VQSLOD score and other annotations from GATK (BaseQRankSum, Dels, FS, HRun, HaplotypeScore, InbreedingCoeff, MQ0, MQRankSum, QD, ReadPosRankSum, culprit) were copied back to the original samtools calls, excluding annotations which already existed in or did not apply to the samtools VCFs (DP and MQ, AC, AN). Each VCF further contained the filters LowQual (a low-quality variant according to GATK) and MinVQSLOD (Variant's VQSLOD score is less than the cutoff). All sites that did not fail these filters were marked as PASS and brought forward to the genotype refinement stage.

Of the 4,030 samples (1,990 TwinsUK and 2,040 ALSPAC) that were submitted for sequencing, 3,910 samples (1,934 TwinsUK and 1,976 ALSPAC) were sequenced and went through the variant calling procedure. Low-quality samples were identified before the genotype refinement by comparing the samples to their GWAS genotypes using about 20,000 sites on chromosome 20. Comparing the raw genotype calls to existing GWAS data, we removed a total of 112 samples (64 TwinsUK and 48 ALSPAC) because of one or more of the following causes: (i) high overall discordance to SNP array data (>3%; 55 TwinsUK and 36 ALSPAC), (ii) heterozygosity rate>3SD from population mean (1 TwinsUK and 1 ALSPAC), suggesting contamination (iii) no SNP array data available for that sample (7 TwinsUK and 0 ALSPAC) and (iv) sample below 4x mean coverage (1 TwinsUK and 11 ALSPAC). Overall, 3,798 samples (1,870 TwinsUK and 1,928 ALSPAC) were brought forward to the genotype refinement step.

The missing- and low-confidence genotypes in the filtered VCFs were filled out through an imputation procedure with BEAGLE 4 (rev909) (ref. 9).

Additional sample-level QC steps were carried out on refined genotypes, leading to the exclusion of additional 17 samples (16 TwinsUK and 1 ALSPAC) because of one or more of the following causes: (i) non-reference discordance (NRD) with GWAS SNP data>5% (12 TwinsUK and 1 ALSPAC), (ii) contamination identified by multiple relations to other samples (13 TwinsUK and 1 ALSPAC), (iii) failed sex check (3 TwinsUK and 0 ALSPAC). To identify contamination we pruned the WGS data to a set of independent SNPs and calculated genome-wide average identity by state between each pair of samples across the two cohorts. Samples were removed if they had >25 relations with IBS>0.125 (a high number of relationships may indicate contamination). The resulting set of contaminated samples corresponded almost completely to the set of samples with NRD>5%. This left a final set of 3,781 samples (1,854 TwinsUK and 1,927 ALSPAC). These VCF files were submitted to the EGA.

### Evaluation of imputation accuracy in the UK10K project

The UK10K final release WGS data of 3,781 samples and 45,492,035 sites was used for creation of haplotype reference WGS data sets. For each chromosome, a summary file was first generated and merged with that of the 1000GP WGS data to identify multi-allelic sites, sites with inconsistent alleles with that of the 1000GP data, and singletons not existing in 1000GP. These sites were excluded to create a new set of VCF files, leaving 26,032,603 sites. The VCF-QUERY tool was used to convert the new VCF files into phased haplotypes and legend files for IMPUTE2. VCF files were converted to binary ped (bed) format and multi-allelic sites excluded, and files were then split into 3MB chunks with ±250 kb flanking regions. SHAPEIT v2 was used to re-phrase the haplotypes. Phasing information from the SHAPEIT output was copied back to the original VCF files, with the phase removed for sites missing due to the MAF cutoff. The phased chunks were then recombined with vcf-phased-join from the vcftools package^15^.

The 1000GP Phase I integrated variant set release (v3) for low-coverage whole-genomes in NCBI build 37 (hg19) coordinates was downloaded from 1000GP FTP site (ftp://ftp.1000genomes.ebi.ac.uk/vol1/ftp/release/20110521/, 23 November 2010 data freezes). This callset includes phased haplotypes for 1,092 individuals and 39,527,072 variants (22 autosome and chromosome X). The haplotypes were inferred from a combination of low-coverage genome sequence data, and they contain SNPs, short INDELs, and large deletions. For each chromosome, a summary file was first generated and merged with that of the UK10K WGS data to identify multi-allelic sites and singletons not polymorphic in UK10K. These sites were excluded to create a new set of VCF files. The final reference panel included all 1,092 samples and 32,449,428 sites. The VCF-QUERY tool was used to convert the new VCF files into phased haplotypes and legend files for IMPUTE2.

A random set of 500 samples passing QC filters was chosen from the TwinsUK (*N*=1,854) and ALSPAC (*N*=1,927) WGS data sets. Genotypes for a total of 13,413 sites (corresponding to the content of the Illumina HumanHap610 SNP-array) on chromosome 20 were extracted from the UK10K WGS data in these 1,000 samples.

For the INCIPE study, 6,200 Caucasian participants were randomly chosen from the lists of registered patients of 62 randomly selected general practitioners based in four geographical areas in the Veneto region, North-eastern Italy^16^. A total of 2,258 samples were genotyped with the HumanCoreExome-12v1-1 platform. A total of 542,585 variants were called using Illumina GENCALL algorithm, 244,594 of which are exonic variants. We conducted further QC evaluation as follows to determine sample and SNP quality. At sample level, we applied the following criteria (i) sample identity was validated through genotyping with an independent typing platform (Sequenom). No samples failed this step. (ii) Twelve pairs of duplicate samples, defined as pairs of individuals with ≥98% concordance genome-wide, were identified. The sample with the lowest call rate of the pair was excluded. (iii) Supplied gender was compared with the genotype-inferred gender (heterozygosity on sample chrX, or is the proportion of chrX SNPs called AB). A Gaussian mixture model was used to find adaptive thresholds Mmax and Fmin (respectively, the maximum male and minimum female heterozygosity on chrX). Overall, 55 samples had chrX heterozygosities that were between Mmax and Fmin, and were excluded from analysis. (iv) Call rate: 90 samples with call rates below 95% were excluded from analysis. (v) 88 samples with autosomal heterozygosity (that is, the proportion of all SNPs with an heterozygous call) score≥3 standard deviations away from the mean were excluded. (vi) Finally, five samples were recommended for exclusion where the normalised magnitude of intensity signal in both channels falls below 0.9. Overall, of the total of 2,258 samples genotyped, 2,145 passed QC filters while 113 samples failed QC filters as indicated above, with some samples failing multiple QC filters. At SNP level, we excluded variants with missingness rate ≥3% or Hardy-Weinberg disequilibrium *P*<1 × 10^−5^. We also checked all alleles to confirm that they are on the positive strand of the human genome by comparing alleles against the 1000G and UK10K data. At the end, there were a total of 346,941 polymorphic variants on autosomes, and 8,822 of those on chromosome 20 were retained for analysis.

*Pseudo-GWAS panel*: for our imputation evaluation, we used 6,300 SNPs on chromosome 20 to mimic a SNP chip in a pseudo-GWAS data set. Before imputation, the two pseudo-GWAS data sets were pre-phased using SHAPEIT v2 (ref. 11) to increase phasing accuracy. The UK10K panel was phased jointly with the entire WGS data set. The INCIPE pseudo-GWAS of 2,145 participants was pre-phased separately.

SHAPEIT v2 was also used for re-phasing the reference haplotypes provided 1000GP and UK10K projects. Per the recommendation of the software, the mean size of the windows in which conditioning haplotypes are defined is set to 0.5MB, instead of 2MB used for pre-phasing GWAS. Owing to the significantly higher number of variants in the WGS data, the re-phasing was conducted by 3MB chunk with 250 kb buffering regions, rather than by whole chromosomes as for the pseudo-GWAS. Imputation was carried out on the same chunks with the same flanking regions.

The following three steps were used to merge two WGS reference panels using IMPUTE2 (version 2.3 and later):

1. Impute the variants that are specific to panel 1 (1000GP) into panel 2 (UK10K).
2. Impute the variants that are specific to panel 2 (UK10K) into panel 1 (1000GP).
3. Treat the imputed haplotypes in both panels (with the union of variants from both) as known (that is, take the best-guess haplotypes) and impute the GWAS cohort in the usual way.

The commands for combining haplotypes with the 1000GP are given in Supplementary Note 3.

Imputation of genotypes from the three phased reference panels (UK10K, 1000GP and UK10K+1000GP) into the two test panels was carried out on chromosome 20 split in 3MB chunks with 250 kb buffer regions. Imputation was performed using standard parameters with IMPUTE2, for example:

./impute2 \

-m genetic_map_chr20_combined_b37.txt \

-h chr20.uk10k.hap.gz \

-l chr20.uk10k.legend.gz \

-known_haps_g chr20.incipe2gwas.known_haps.gz \

-k_hap 10000 \

-int 3e6 6e6 \

-Ne 20000 \

-buffer 250 \

-use_prephased_g \

-o_gz \

-o chr20.01.incipe2gwas.uk10kRef.impute2

In Fig. 1a,d, the accuracy of imputed variants was calculated as the Pearson correlation coefficient (*r*^2^) between imputed genotype dosages in (0–2) and masked sequence genotypes in (0,1,2). The results were stratified into non-overlapping MAF bins for plotting. In Fig. 1b,c, the numbers of variants in different imputation accuracy bins were estimated via the expected *r*^2^ (‘info') metric produced by IMPUTE2 (ref. 13). As discussed in the main text, this metric is biased upward relative to the true *r*^2^, so the numbers of high-confidence variants in these figures should be interpreted as upper bounds.

## 

## Additional information

**Accession Codes:** UK10K reference haplotypes are available from the European Genome-phenome archive under the accession codes EGAS00001000713 (EGA study) and EGAD00001000776 (EGA dataset) under managed access conditions (http://www.uk10k.org/data_access).

**How to cite this article:** Huang, J. *et al*. Improved imputation of low frequency and rare variants using the UK10K haplotype reference panel. *Nat. Commun.* 6:8111 doi: 10.1038/ncomms9111 (2015).

## Supplementary Material

Supplementary Information Supplementary Figures 1-6, Supplementary Table 1, Supplementary Notes 1-3, and Supplementary References

## Figures and Tables

**Table 1:** Descriptives for the UK10K and 1000GP reference panels used for imputation.

	**UK10K**	**1000GP(Phase 1 v3)**	**Combined**	**Overlap**
N samples (% European)	3,781 (100%)	1,092 (34.7%)	4,873	—
N total sites in final release	45,492,035	39,527,072	—	
N total sites after filtering*	26,032,603	32,449,428	42,359,694	16,122,337
**Autosome SNPs**	23,411,635	29,797,220	38,238,102	14,970,753
**Autosome INDELs**	1,698,262	1,370,819	2,407,858	661,223
**Chr X SNPs**	858,380	1,223,328	1,612,230	469,478
**Chr X INDELs**	64,326	58,061	101,504	20,883

^*^For UK10K, the following sites were excluded: 18,180,633 singletons that do not exist in 1000GP, 1,064,168 multi-allelic sites and 214,631 mis-matched alleles sites. For 1000GP, the following sites were excluded: 7,053,246 singletons that do not exist in UK10K, 23,932 sites with a SNP and an INDEL at the same position and 443 within large structural deletions. The bold indicates that these four categories of variants are subsets of the N total sites after filtering.
