# Supplementary material for: Improved imputation of low-frequency and rare variants using the UK10K haplotype reference panel
Source: Nat Commun. 2015 Sep 14;6:8111. doi: 10.1038/ncomms9111 (PMC4579394; doi:10.1038/ncomms9111)
Supplement: Supplementary Information — Supplementary Figures 1-6, Supplementary Table 1, Supplementary Notes 1-3, and Supplementary References [file ncomms9111-s1.pdf]

Supplementary Figures

Supplementary Figure 1. Diagram illustrating the novel software functionality for merging haplotype panels in IMPUTE2.

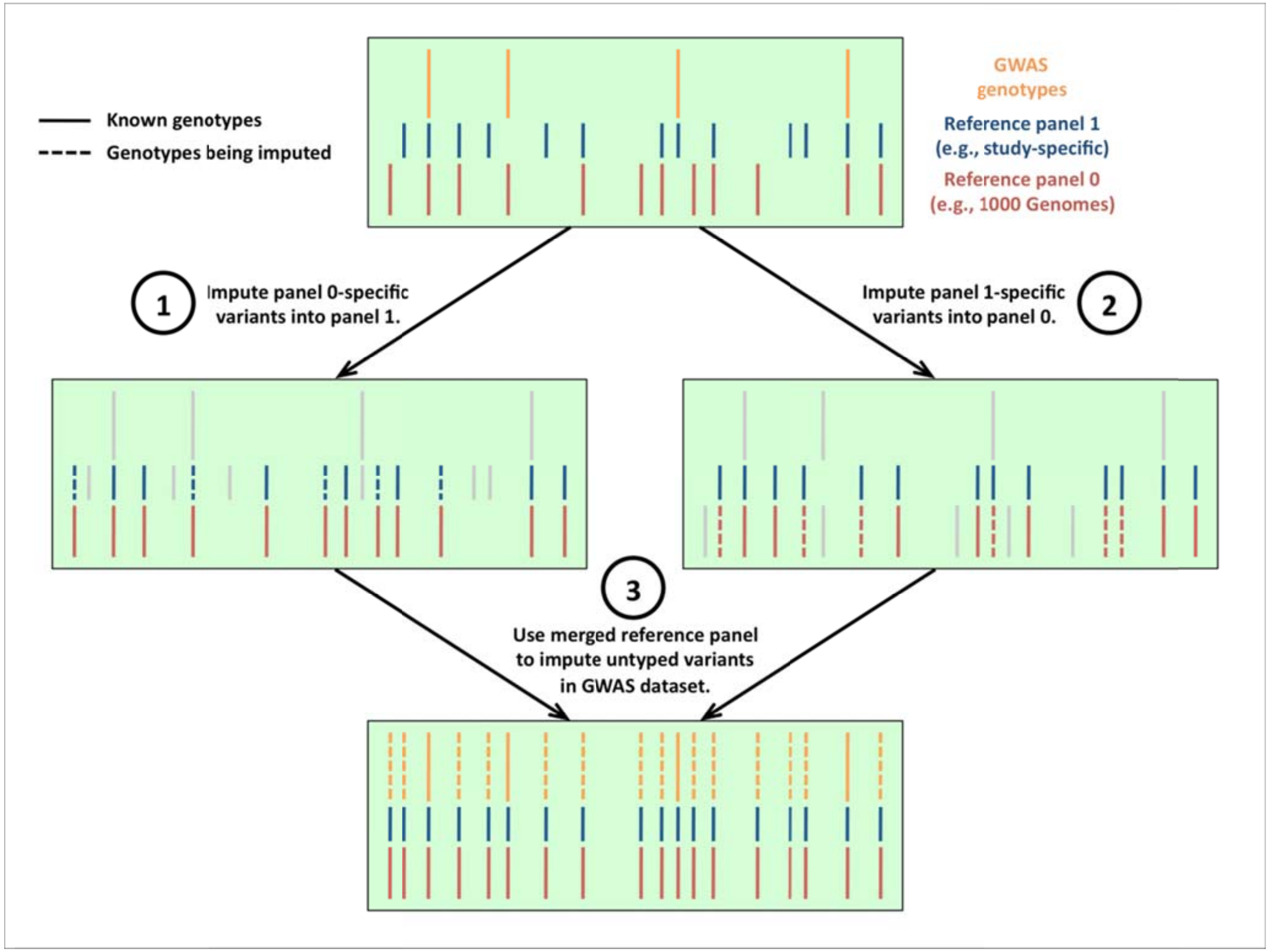

Supplementary Figure 2. Flowchart describing the strategy for imputation evaluation in this study.

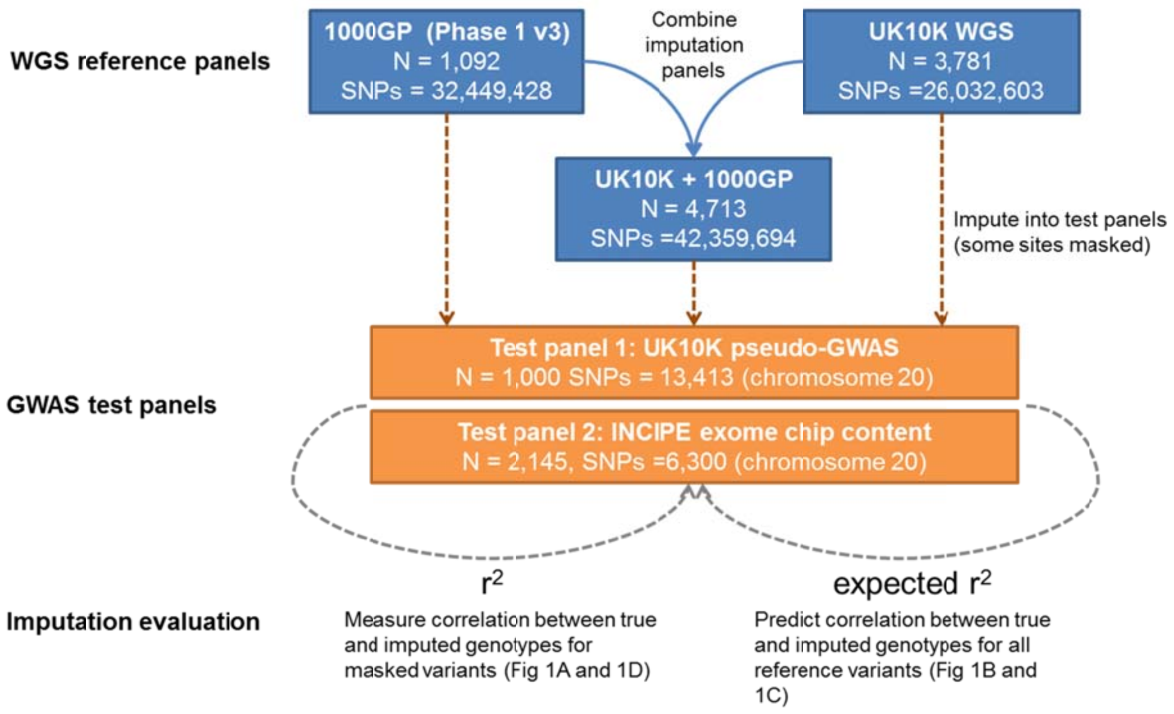

Supplementary Figure 3. Imputation performance of different reference panels

Imputation accuracy in the UK10K pseudo-GWAS test panel using reference panels from 1000GP (black), UK10K (blue), and UK10K+1000GP (red) across all MAFs. The "original" UK10K reference panel (dotted blue line) was produced by standard genotype refinement of low-coverage sequencing data, while the "rephased" reference panel (solid blue line) was produced by running SHAPEIT2 on the genotypes called by BEAGLE to improve haplotype accuracy. The rephased UK10K panel was combined with the 1000GP panel to produce the UK10K+1000GP panel.

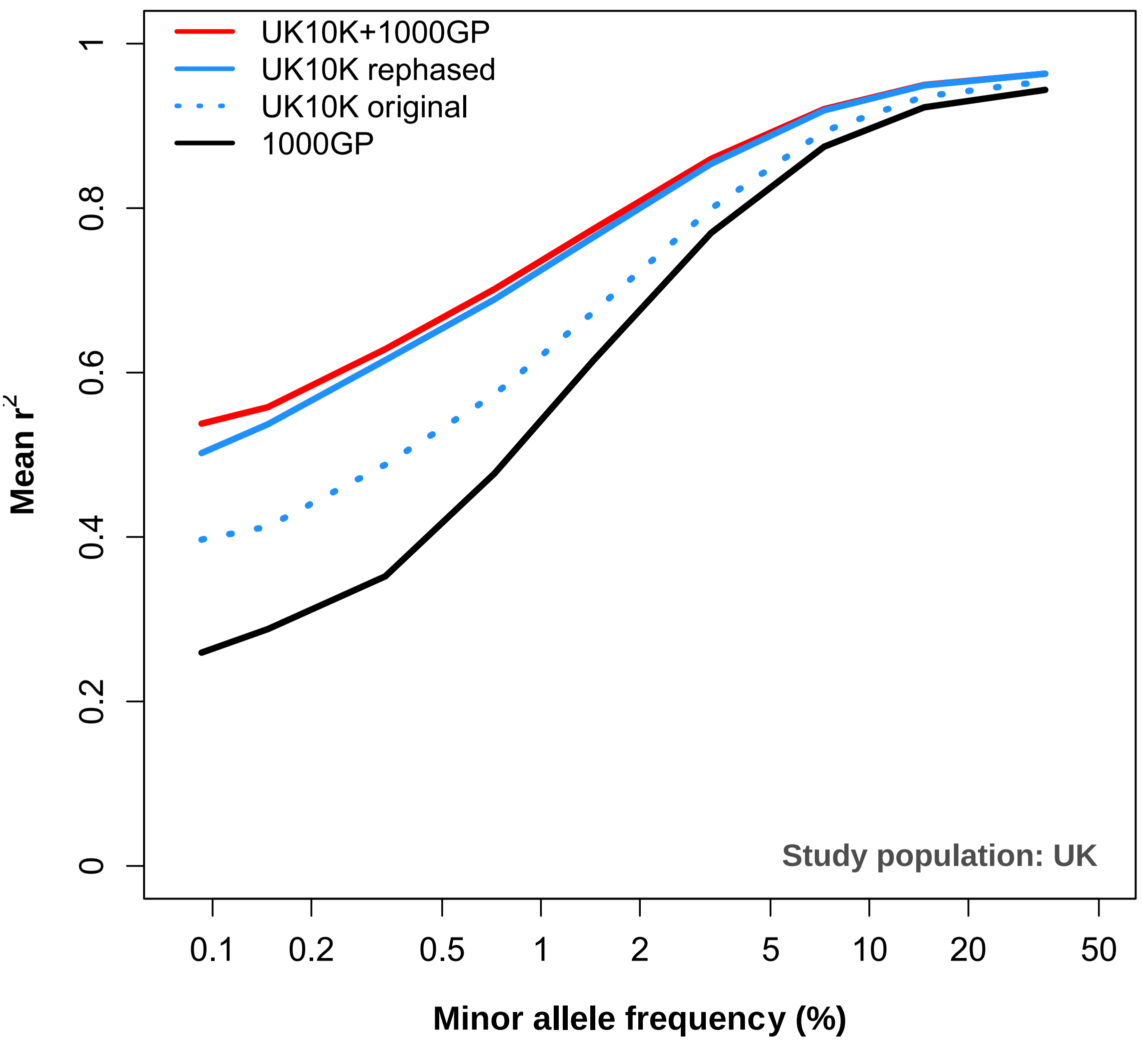

Supplementary Figure 4. SNP-wise imputation accuracy with a Hamming distance approximation.

SNPs with MAF<5% in the INCIPE pseudo-GWAS panel were imputed with the UK10K reference panel under two different IMPUTE2 settings: one that used a Hamming distance approximation to choose a customized subset of 500 reference haplotypes when imputing each study haplotype (x-axis; mean r^2=0.27), and one that used all available reference haplotypes with no approximation (y-axis; mean r^2=0.33). The red point highlights a rare SNP that is examined in detail in the supplementary text and figures.

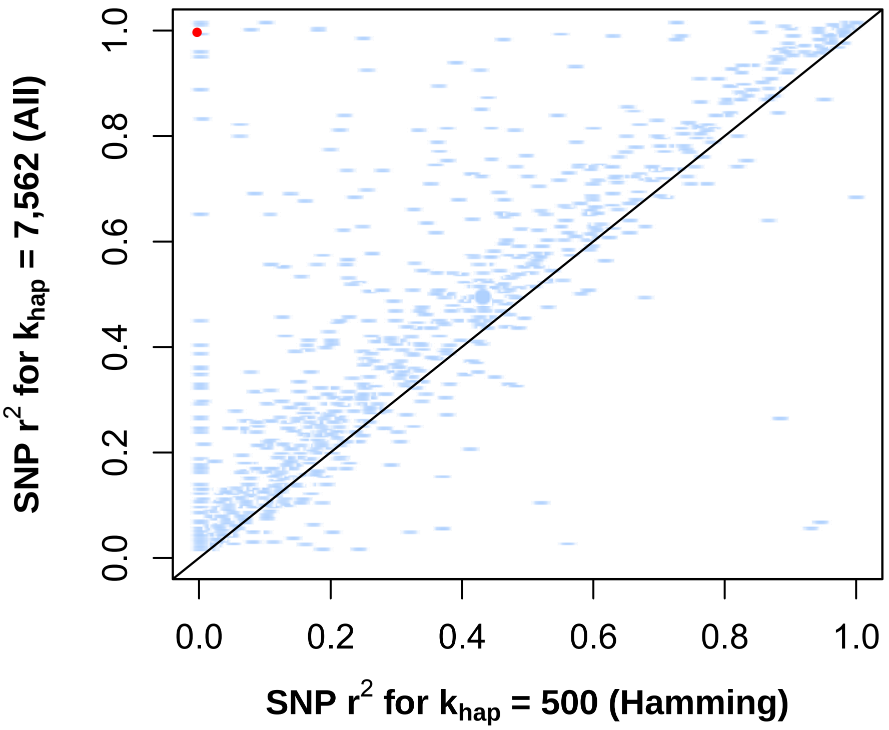

Supplementary Figure 5. Illustration of reference states (haplotypes) copied by IMPUTE2 when imputing INCIPE

INCIPE pseudo-GWAS haplotypes were imputed from the UK10K reference panel in a 3Mb region on chromosome 20. Points at each position on the chromosome (x-axis) represent reference haplotypes that were copied with marginal (per-site) posterior probabilities of at least 0.01 when using the full UK10K reference panel (7,562 haplotypes). Copied reference haplotypes are ordered on the y-axis by the position at which they first surpassed this threshold. The location of the SNP coloured red in **Supplementary Figure 4** is marked by a vertical red line, and points belonging to the haplotype that carries this variant are also coloured red. Subsets of reference states selected by different approximations are marked by dotted blue lines. **(A)** Reference states selected with k_{hap}=500 under a Hamming distance approximation. Of the 103 copied states in this plot, 25 (24%) were chosen under this approximation. **(B)** Reference states selected with k_{hap}=500 under a tract sharing approximation. Of the 103 copied states in this plot, 96 (93%) were chosen under this approximation.

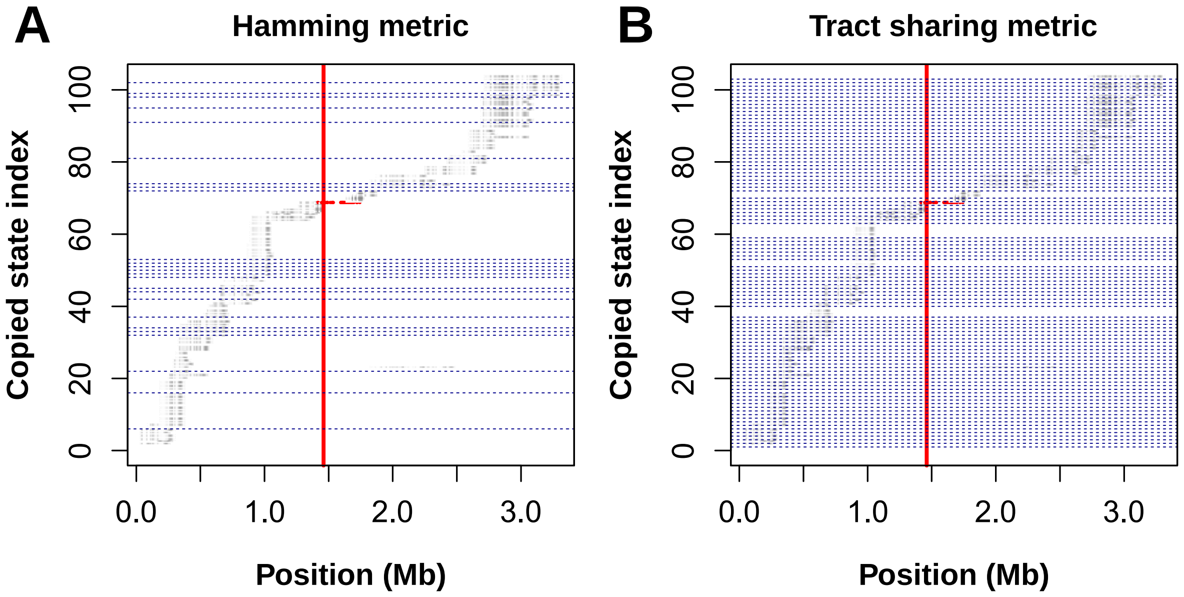

Supplementary Figure 6. SNP-wise imputation accuracy with a tract sharing approximation.

SNPs with MAF<5% in the INCIPE pseudo-GWAS panel were imputed with the UK10K reference panel using either all available UK10K haplotypes or a customized subset of UK10K haplotypes chosen by a tract sharing approximation. The rare SNP highlighted in red in **Supplementary Figure 4** is also shown in red here. **(A)** Comparison of imputation accuracy with k_{hap}=500 (x-axis; mean r^2=0.32) against accuracy with the full reference panel (y-axis; mean r^2=0.33). **(B)** Comparison of imputation accuracy with k_{hap}=60 (x-axis; mean r^2=0.30) against accuracy with the full reference panel (y-axis; mean r^2=0.33).

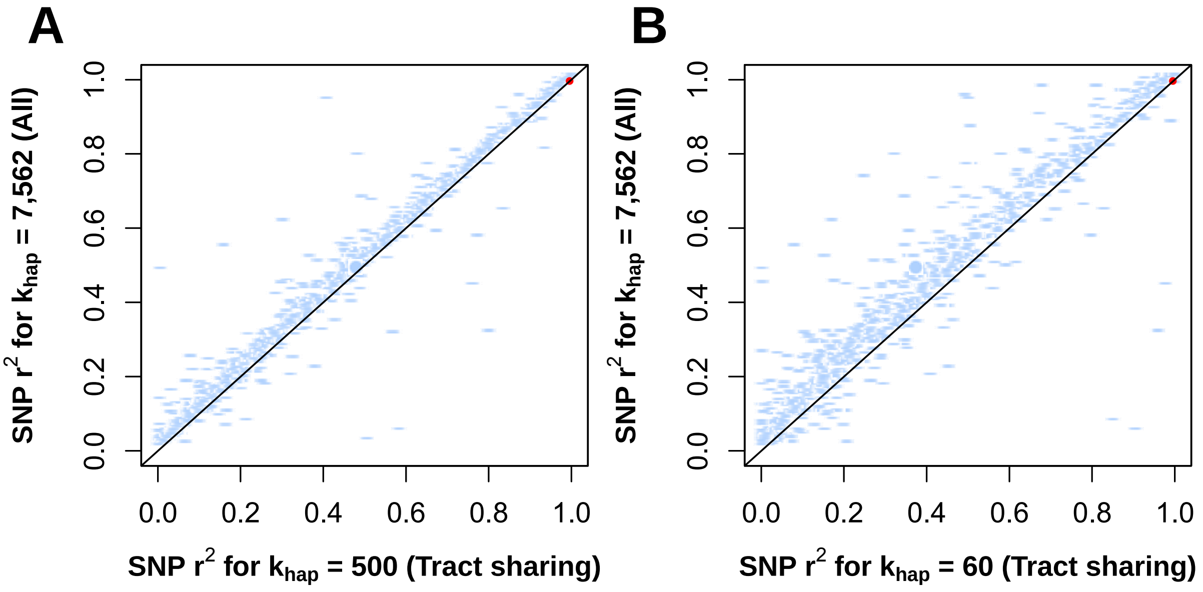

Supplementary Table

Supplementary Table 1. Imputation results for different reference panels.

Numeric values of imputation r^2 are given for SNPs and IN/DELs within each allele frequency bin; the UK10K pseudo-GWAS is the imputation target

**MAF bin**	**0.001**	**0.002**	**0.005**	**0.01**	**0.02**	**0.05**	**0.1**	**0.2**	**0.5**
UK10K+1000GP	0.538	0.558	0.629	0.702	0.774	0.860	0.921	0.950	0.963
UK10K rephased	0.502	0.537	0.615	0.689	0.764	0.854	0.919	0.949	0.964
UK10K original	0.397	0.413	0.488	0.573	0.673	0.799	0.894	0.936	0.954
1000GP	0.259	0.288	0.352	0.477	0.615	0.770	0.875	0.923	0.944

Supplementary Notes

Supplementary Note 1. Imputation strategy and novel software functionality for merging WGS datasets

Genotype imputation is now widely used in GWAS to boost power, carry out fine-mapping and facilitate meta-analysis[1]. Usually imputation is carried out using a single haplotype reference panel, such as those produced by the HapMap project or the 1000 Genomes Project. We have developed a new option in the Impute2 software[2,3] that allows two sets of haplotypes to be combined to form a single set of haplotypes at the union set of sites. Imputation into GWAS samples can then be carried out using this combined panel. This method can be used to combine two sets of haplotypes from two distinct population cohorts, such as UK10K and 1000 Genomes, as we have done in this paper. Alternatively, it may be that a particular study has sequenced specific individuals with high relevance to the GWAS, and wish to combine that set of haplotypes with one of the publicly available haplotype sets.

The main difficulty in combining reference panels is that some sites will only have data in one or other of the panels. This maybe because the site is monomorphic for the reference allele in the cohort, in which case the site is unlikely to have been `called' from the sequencing. However, the site may also be polymorphic and may not have been called due to low-coverage of the non-reference allele, or due to cohort specific site filtering that removed the site from consideration.

We use the Li and Stephens[4] hidden Markov model to impute the unobserved alleles in each panel using the other panel. Since each dataset is haploid the calculations involved are efficient. The methods build upon the pre-phasing imputation machinery already within Impute2. The scheme that we use to carry out imputation is shown in **Supplementary Figure 1.**

We denote the two haplotype reference panels as panel 0 and panel 1. The top part of the figure shows the combined datasets. In the diagrams a column is a site and a row is an individual. Observed genotypes in the two reference panels are coloured red and blue

respectively. Observed genotypes in the GWAS samples are coloured orange. The top figure makes it clear that that some sites only have observed genotypes in some of the datasets.

We impute the untyped variants in three steps:

1. Impute the variants that are specific to Panel 0 (red) into Panel 1 (blue). Variants shown in grey do not inform the imputation.
2. Impute the variants that are specific to Panel 1 (blue) into Panel 0 (red). Variants shown in grey do not inform the imputation.
3. Now that we have imputed the two reference panels up to the union of their variants, treat the imputed haplotypes as known (*i.e.*, take the best-guess haplotypes) and impute the GWAS cohort in the usual way.

Our implementation allows for the use of unphased or pre-phased GWAS samples. In addition, Impute2 outputs a file containing a merged haplotype reference panel that can be used for future imputation without repeating this step. This new functionality is available in IMPUTE2 v2.3.1 at https://mathgen.stats.ox.ac.uk/impute/impute_v2.html.

Supplementary Note 2. Novel imputation approximation based on haplotype tract sharing

Background and motivation

Genotype imputation in GWAS has always been a computationally intensive task. Recent developments like pre-phasing have greatly reduced the computational cost of imputation, but growing reference panels continue to challenge existing methods. As we were conducting the analyses for this manuscript, we evaluated an approximation developed by Howie et al. (2011) to reduce computing times for GWAS imputation. This approach uses a Hamming distance metric to choose a different subset of k_{hap} reference haplotypes for each GWAS haplotype; if this subset includes the most informative reference haplotypes, it can speed up the imputation calculations without sacrificing much accuracy. The cost of imputation with pre-phased GWAS data scales linearly with the number of reference haplotypes N, so the speedup expected from this approximation is roughly N / k_{hap} after accounting for the overhead of reading in a large data set.

In an experiment that used the INCIPE pseudo-GWAS panel and the UK10K reference panel, we compared the results of running IMPUTE2 with k_{hap}=500 and with no approximation (effectively k_{hap}=7,562, the number of haplotypes in the full UK10K panel). The results for SNPs with MAF<5% are shown in **Supplementary Figure 4**. The full reference panel (y-axis) produced better accuracy for most SNPs, but the differences were generally modest – overall, the mean r^2 was 0.27 for k_{hap}=500 and 0.33 with no approximation. However, a subset of SNPs were imputed very poorly (r^2<0.2) with the Hamming distance approximation and very well (r^2>0.8) with the full panel. We decided to investigate further to understand where the approximation was breaking down, focusing initially on a singleton SNP (frequency 0.1%) among the masked and imputed INCIPE SNPs (position 1,460,491 on chromosome 20; shown in red in **Supplementary Figure 4** and subsequent figures).

Understanding limitations of the Hamming approximation

To better understand why the Hamming distance approximation failed to successfully impute the rare SNP highlighted above, we examined the state-copying probabilities generated by IMPUTE2 when no approximation was used with the UK10K reference panel. These probabilities are calculated at each site that is shared between a GWAS data set and a

reference panel. At a given site, the reference haplotypes ("copying states") with the largest probabilities contribute the most to the imputation.

Supplementary Figure 5 provides a visual depiction of how the copied reference states changed across a 3MB region of chromosome 20 when the INCIPE haplotype carrying the singleton allele of interest was being imputed. Walking from left to right across the region, each reference haplotype that was copied with a per-site (marginal) probability of 0.01 or greater was added to a list of unique copied states, which were assigned to consecutive positions on the y-axis. There is a grey point on the plot for each reference haplotype that surpassed this threshold at a given SNP site (x-axis location); most points blur together since there are many on the plot. The location of the SNP of interest is shown as a vertical red line, and the points that correspond to the UK10K haplotype that carries the variant allele are also coloured red.

Intuitively, **Supplementary Figure 5** gives a sense of where the imputation model chose to copy different reference haplotypes as it scanned the region. The probabilities that generated this plot are based on the full UK10K reference panel, so the 103 copying states represented in this plot are among the most important for imputing this GWAS haplotype – we would hope than an approximation would choose many of these reference states from the full set of 7,562 UK10K haplotypes.

Supplementary Figure 5A shows which states were chosen by the Hamming distance metric with k_{hap}=500. Each state chosen by this approximation is shown as a horizontal dotted line; 25 of the 500 selected states were among the 103 states in this plot, but these did not include the haplotype carrying the highlighted rare variant, which is why it was not successfully imputed by the Hamming method.

A notable feature of this plot is that the copied states change frequently along the region, which is a consequence of the high recombination rate in this region (average of 3.5 cM/Mb). It can also be seen that the shared haplotype tract of interest, shown as a row of red dots, is distinctive and short: within the range of the red dots, this haplotype is often the only one with a meaningful copying probability, yet the shared tract is only ~300kb long (many alleles at 0.1% frequency reside on longer haplotype backgrounds). There is a clear

signal of haplotype sharing to be found here, but it is not easy to detect via region-wide metrics like Hamming distance.

Observations like this led us to develop a new approximation that focuses on capturing the shared reference haplotype tracts around each site in a study haplotype, rather than averaging these out with region-wide metrics. Our goal was to capture the same kind of information used by methods like MVNcall[5] for one site at a time, but to do so in a way that produces an ensemble of k_{hap} haplotypes that can be used to impute an entire region, analogous to the current Hamming distance approach used by IMPUTE2.

A novel tract sharing approximation:

The goal behind our new approximation is to ensure that each site in a study haplotype has the opportunity to copy the reference haplotype with the longest shared tract of allelic identity. If this goal can be fulfilled with fewer than k_{hap} reference haplotypes, we continue adding haplotypes with shorter shared tracts until k_{hap} unique states have been selected. This approach aims to capture local copying information while allowing a user to control the computational costs via k_{hap}, as is currently done with the Hamming distance method.

Our algorithm works as follows, from the point of view of a single GWAS haplotype:

1. For each reference haplotype, identify sets of contiguous sites that show no allele mismatches with the study haplotype; store these shared haplotype tracts for each reference haplotype.
2. At each site, generate a hash table whose keys are shared tract lengths (in genetic map units) and whose values are indices of the corresponding reference haplotypes. A given key can map to multiple values.
3. At each site, use the hash table created in the previous step to generate a list of reference haplotype indices ranked in descending order of shared tract length. Ties are broken at random.
4. Add the top-ranked haplotype index at each site to a list of unique reference haplotype indices; these states are marked for copying by the current study haplotype.

5. Go to the next-ranked haplotype index ("level") and repeat Step 4 until k_{hap} distinct reference haplotypes have been identified. If the number of selected haplotypes exceeds k_{hap} at a particular level, choose a random subset of the reference indices at that level such that the total number of selected haplotypes is k_{hap}.

Supplementary Figure 5B shows that this algorithm is much more effective than the region-wide Hamming metric at identifying reference haplotypes with the highest local copying probabilities: whereas the Hamming method selected 25/103 (24%) of the most important reference states at k_{hap}=500, the tract sharing method selected 96/103 (93%) of these states. These results suggest that our new approximation may better reflect the behaviour of the IMPUTE2 model with a full reference panel, which should lead to more accurate imputation.

Computational burden and accuracy of tract sharing approximation

The computational cost of imputing a study haplotype with the Hamming distance approximation is *O(MN)*, where *M* is the number of sites shared between the study and reference panels and *N* is the number of reference haplotypes. Our new tract length approximation takes roughly four times longer since it scans the sites in a region multiple times, but it is still linear in *M* and *N*. The Hamming distance approximation accounts for less than 1% of a typical imputation run (as determined by profiling the IMPUTE2 C++ code when imputing the INCIPE pseudo-GWAS with the UK10K reference panel), so switching to the tract sharing approximation leads to only a small increase in total run times – typically less than 5% in the benchmark experiments we conducted.

To confirm that the tract sharing approximation improves imputation accuracy, we repeated the analysis from **Supplementary Figure 4** (SNPs with MAF<5% imputed with the INCIPE pseudo-GWAS panel and the UK10K reference panel). **Supplementary Figure 6A** shows that the new approximation with k_{hap}=500 provides essentially the same accuracy as using the entire UK10K reference panel: the mean r^2 values in this analysis were 0.32 and 0.33, respectively, and none of the SNPs imputed well (r^2>0.8) by the full reference panel were missed when using the approximation – this includes the rare SNP that was previously imputed poorly by the Hamming distance method (red dot). To see if we could push this approach even further, we also ran the tract sharing approximation with k_{hap}=60 (**Supplementary Figure 6B**). The accuracy suffered a bit at this setting (mean r^2=0.30), but

the results were still better than the analysis with k_{hap}=500 under the Hamming metric, and again there were few major discrepancies between the results with this approximation versus the full reference panel.

Conclusions

In summary, our new tract sharing approximation has a similar computational cost to the Hamming distance approximation of [3], but it is better at maintaining imputation accuracy for low-frequency and rare SNPs. We believe that this will be a useful approach as imputation reference panels continue to grow.

Supplementary Note 3. Re-phasing and imputation commands.

These are the command options for imputation using the combined UK10K+1000GP panel in IMPUTE2, using as an example for one region of chromosome 20.

1. Phase UK10K WGS with SHAPEIT v2

shapeit --thread 8 --window 0.5 --states 200 --effective-size 11418 –B chr20.01--input-map genetic_map_chr20_combined_b37.txt --output-log chr20.shapeit --output-max chr20.hap.gz chr20.sample

2. Merge WGS reference panel

impute2 -allow_large_regions -m genetic_map_chr20_combined_b37.txt -h 1kg/chr20.01.shapeit.hap.gz uk10k/chr20.01.shapeit.hap.gz -l 1kg/chr20.01.shapeit.legend.gz uk10k/chr1.01.shapeit.legend.gz -merge_ref_panels -merge_ref_panels_output_ref chr20.01.shapeit -int 28590 3028590 -Ne 20000 -buffer 250 -include_buffer_in_output

3. Pre-phase UK10K-Cohort GWAS

shapeit --thread 8 --window 2 --states 200 --effective-size 11418 -B chr20.01 --input-map genetic_map_chr20_combined_b37.txt --output-log chr20.01.shapeit --output-max chr20.01.hap.gz chr20.01.sample

4. Imputation

impute2 -allow_large_regions -m genetic_map_chr20_combined_b37.txt -h chr20.01.shapeit.hap.gz -l chr20.01.shapeit.legend.gz -known_haps_g chr20.hap.gz -sample_g chr20.sample -exclude_samples_g uk10k.sample.ids -use_prephased_g -int 28590 3028590 -Ne 20000 -buffer 250 -o chr20.01.gen

Supplementary references

1. Marchini, J. & Howie, B. Genotype imputation for genome-wide association studies. *Nat Rev Genet* **11**, 499-511 (2010).
2. Howie, B.N., Donnelly, P. & Marchini, J. A flexible and accurate genotype imputation method for the next generation of genome-wide association studies. *PLoS Genet* **5**, e1000529 (2009).
3. Howie, B., Marchini, J. & Stephens, M. Genotype imputation with thousands of genomes. *G3 (Bethesda)* **1**, 457-70 (2011).
4. Li, N. & Stephens, M. Modeling linkage disequilibrium and identifying recombination hotspots using single-nucleotide polymorphism data. *Genetics* **165**, 2213-33 (2003).
5. Menelaou, A. & Marchini, J. Genotype calling and phasing using next-generation sequencing reads and a haplotype scaffold. *Bioinformatics* **29**, 84-91 (2013).
